# Cross-Calibration of GE Healthcare Lunar Prodigy and iDXA Dual-Energy X-Ray Densitometers for Bone Mineral Measurements

**DOI:** 10.1155/2016/1424582

**Published:** 2016-04-27

**Authors:** J. Saarelainen, M. Hakulinen, T. Rikkonen, H. Kröger, M. Tuppurainen, H. Koivumaa-Honkanen, R. Honkanen, M. Hujo, J. S. Jurvelin

**Affiliations:** ^1^Kuopio Musculoskeletal Research Unit (KMRU), Surgery, Institute of Clinical Medicine, University of Eastern Finland, 70211 Kuopio, Finland; ^2^Department of Applied Physics, University of Eastern Finland, 70211 Kuopio, Finland; ^3^Department of Clinical Physiology and Nuclear Medicine, Kuopio University Hospital, 70029 Kuopio, Finland; ^4^Department of Orthopaedics, Traumatology and Hand Surgery, Kuopio University Hospital, 70029 Kuopio, Finland; ^5^Department of Obstetrics and Gynaecology, Kuopio University Hospital, 70029 Kuopio, Finland; ^6^Institute of Clinical Medicine, Psychiatry, University of Eastern Finland, 70211 Kuopio, Finland; ^7^Departments of Psychiatry, Kuopio University Hospital, 70029 Kuopio, Finland; ^8^South-Savonia Hospital District, 50520 Mikkeli, Finland; ^9^North Karelia Central Hospital, 80210 Joensuu, Finland; ^10^SOSTERI, 57120 Savonlinna, Finland; ^11^SOTE, 74101 Iisalmi, Finland; ^12^Lapland Hospital District, 96101 Rovaniemi, Finland; ^13^Clinic of Child Psychiatry, University Hospital of Oulu, 90029 Oulu, Finland; ^14^School of Computing, University of Eastern Finland, 70211 Kuopio, Finland; ^15^Diagnostic Imaging Centre, Kuopio University Hospital, 70029 Kuopio, Finland

## Abstract

In long-term prospective studies, dual-energy X-ray absorptiometry (DXA) devices need to be inevitably changed. It is essential to assess whether systematic differences will exist between measurements with the new and old device. A group of female volunteers (21–72 years) underwent anteroposterior lumbar spine L2–L4 (*n* = 72), proximal femur (*n* = 72), and total body (*n* = 62) measurements with the Prodigy and the iDXA scanners at the same visit. The bone mineral density (BMD) measurements with these two scanners showed a high linear association at all tested sites (*r* = 0.962–0.995; *p* < 0.0001). The average iDXA BMD values were 1.5%, 0.5%, and 0.9% higher than those of Prodigy for lumbar spine (L2–L4) (*p* < 0.0001), femoral neck (*p* = 0.048), and total hip (*p* < 0.0001), respectively. Total body BMD values measured with the iDXA were −1.3% lower (*p* < 0.0001) than those measured with the Prodigy. For total body, lumbar spine, and femoral neck, the BMD differences as measured with these two devices were independent of subject height and weight. Linear correction equations were developed to ensure comparability of BMD measurements obtained with both DXA scanners. Importantly, use of equations from previous studies would have increased the discrepancy between these particular DXA scanners, especially at hip and at spine.

## 1. Introduction

Reliable follow-up of bone mineral density (BMD) by dual-energy X-ray absorptiometry (DXA) scans is essential both in clinical practice and in medical research. However, aging or defective DXA technology may compromise reliability of subsequent DXA measurements and, thus, require change of machinery. International Society for Clinical Densitometry (ISCD) recommends an* in vivo* cross-calibration procedure if the old DXA system is replaced with a different DXA model regardless of whether it is from the same or different manufacturer [1]. Cross-calibration is important because systematic differences between the instruments may even exceed the annual biological BMD changes [1]. Some cross-calibration studies have suggested that inclusion of anthropometrical measurements may improve agreement between the DXA densitometers [2, 3]. Unfortunately, discrepancies exist among the previous studies investigating the agreement of BMD measurements between GE Healthcare Lunar Prodigy and iDXA devices [4–8].

The Kuopio Osteoporosis Risk Factor and Prevention (OSTPRE) Study in Eastern Finland, started in 1989 [9], evaluates also long-term BMD changes in a Caucasian female population born between 1932 and 1941 using DXA densitometry repeated at 5-year intervals. At present, its 25th-year measurements are currently ongoing. Until now four different DXA scanners from the same manufacturer have been used.

In the present study, we (1) evaluate the agreement of bone mineral measurements between GE Healthcare Lunar (Madison, WI, USA) Prodigy and iDXA narrow-angle fan beam densitometers; (2) compare cross-calibration results acquired from human and phantom data; (3) calculate potential correction coefficients for Prodigy results to match with those of iDXA; and (4) evaluate the effect of body anthropometry parameters on the agreement of BMD measurements between the instruments.

## 2. Materials and Methods

Study subjects (*n* = 72, aged 21–72 years) of over 20 years of age were recruited from the volunteers in University of Eastern Finland. Exclusion criteria included pregnancy and bilateral hip prostheses. Total body DXA could be conducted for a smaller group of subjects (*n* = 62) due to technical limitations, that is, too narrow scan table in Prodigy for one severely obese (126 kg) subject and two subjects with metal in their body, as well as due to reluctance of some subjects for total body measurements.

The Prodigy scanner, equipped with a narrow fan beam at an angle of 4.5° and orientated parallel to the long axis of the body, applies constant peak X-ray energy at 76 kV and a current of 3 mA. Further, a Samarium K-edge filter produces energies at 38 and 70 kV [5]. The Prodigy system employs 16 Direct-Digital high-definition (HD) detectors, made of energy sensitive cadmium zinc telluride (CZT), 5 cm long, and they allow rapid photon counting. The iDXA uses a peak X-ray energy of 100 kV as well as an array of sixty-four Direct-Digital CZT-HD detectors, which eliminate dead space between the detectors, thereby creating a high resolution image and improvement of precision for the scan [5]. The improved image resolution of iDXA comes at the cost of a slightly higher effective radiation dose, as compared to Prodigy. In both devices, however, typical radiation dose for a subject is less than 10 *μ*Sv.

Left proximal femur (total hip, femoral neck, shaft, Ward's triangle, and trochanter) and anteroposterior (AP) lumbar spine (L2–L4, L2-L3, and L3-L4) of 72 women were scanned by using both GE Healthcare Lunar Prodigy (software version 11.4) and iDXA (software version 14.0) narrow-angle fan beam densitometers. In addition, 62 women completed also total body BMD (g/cm^2^), bone mineral content (BMC, g), and bone area (cm^2^) measurements during the same visit between June and September of 2012. In order to minimize the potential operator bias all scans on both devices were performed in the same room by two experienced nurses. Subjects were carefully repositioned between the scans to minimize errors that could be related to changes in the measurement geometry [10]. For analysis, the automatic edge detection was always used; however, all BMD analyses were thoroughly checked for errors and manually corrected if needed. The GE Healthcare Lunar algorithm that automatically finds the area of the lowest BMD in proximal femur, that is, Ward's triangle, was used in our study. Subject weight and height were measured during each visit. All study subjects provided informed consent and the research protocol was approved by the ethics committee of Kuopio University Hospital (KUH). During the study period, quality control scans on spine phantom (L2–L4) were performed along the guidelines of the manufacturer.

## 3. Statistics

To reveal the association and agreement between the measurements of two densitometers the data were analyzed by using the linear regression analysis, Deming regression, paired *t*-test, Pearson's correlation analysis, and Bland and Altman analysis [11]. If the assumption of normal distribution was violated, the nonparametric Wilcoxon signed-rank test was used to analyze the differences between devices (e.g., Ward's triangle BMD, BMC, and area). For BMD_Prodigy_ versus BMD_iDXA_ scatter plots the statistical significance of the intercept of each regression line was tested. If the intercept was not different from zero then the regression analysis was repeated with the intercept forced through the origin [1]. The effect of body height, weight, and mass index (BMI, kg/m^2^) on the cross-calibration was studied using the stepwise multivariate linear regression analysis. Height and BMI as well as weight and BMI were not included in the same model due to multicollinearity based on variance inflation functions and tolerance statistics. The accuracy of corrections, obtained with a regression line, was expressed as the standard error of the estimates (SEE). The Bland and Altman method was used to evaluate the bias in results between the devices [11].

Hologic anthropometric lumbar spine phantom, European Spine Phantom (ESP), and GE Healthcare Lunar aluminum spine phantom [12, 13] were scanned 10 times during a period of one week to calculate the short-term precision error (coefficients of variation, CV% = (SD/mean) × 100%) of the instruments [14].

Statistical analyses were performed with the R Statistical Software (Foundation for Statistical Computing, Vienna, Austria) and with the Statistical Package for Social Sciences (IBM SPSS Statistics for Windows, IBM Corp., Armonk, NY, USA, version 19.0). A *p* value below 0.05 was considered to be statistically significant.

## 4. Results

*In vitro* scans of the three phantoms indicated that iDXA measured 0.4% and 1.0% higher BMD values with Lunar and ESP phantoms compared to Prodigy, respectively, whereas iDXA measured −0.4% lower BMD values with Hologic phantom compared to Prodigy (Table 1). Short-term precision (repeated phantom measurements) of BMD values* in vitro* was slightly lower (i.e., CV% higher) when measured with Prodigy (0.34–0.50%) than with iDXA (0.13–0.42%).

**Figure 1:**
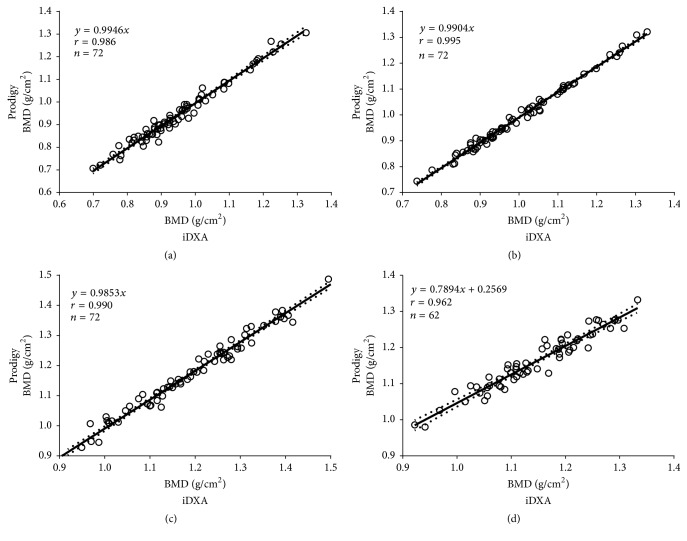
Linear correlations and 95% confidence intervals between bone mineral density (BMD) values measured by the GE Healthcare Lunar Prodigy and iDXA. (a) Femoral neck, (b) total hip, (c) lumbar spine (L2–L4), and (d) total body of the study subjects. Linear correlation (*r*) of BMD values between DXA devices was high at spine and at hip, whereas the association was slightly lower at total body BMD.

**Figure 2:**
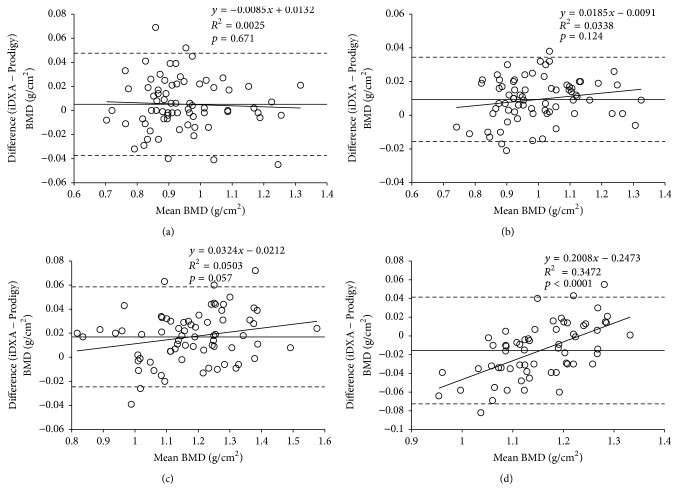
Bland and Altman analysis: agreement between GE Healthcare Lunar Prodigy and iDXA scans. The difference in BMD values between two scans as a function of the mean value for the two DXA scanners at the (a) femoral neck, (b) total hip, (c) lumbar spine (L2–L4), and (d) total body. Mean difference (*d*) = straight line; limits of agreement (*d* ± 1.96 × SD) = dashed lines. *N* = 72 ((a) to (c)); *N* = 62 (d). The total body BMD difference was strongly dependent on mean BMD: at high BMD values iDXA showed significantly higher values than Prodigy, whereas at low BMD values the opposite was found. The hip and spine BMD difference was less dependent on the mean BMD values.

Age and anthropometrical variables were recorded in subjects within the cross-calibration sample (Table 2). BMD scatter plots (Prodigy versus iDXA) showed a close linear relationship over the entire range of BMD values for the spinal, femoral neck, total hip, and total body scans (*r* = 0.962–0.995, *p* < 0.0001) (Figure 1).* In vivo *BMD values of iDXA were, as compared to Prodigy, 1.5% (0.017 g/cm^2^) higher at the lumbar spine (L2–L4), 0.5% (0.005 g/cm^2^) higher at the femoral neck, and 0.9% (0.009 g/cm^2^) higher at the total hip (Table 3). In contrast, total body BMD values by iDXA were −1.3% (−0.016 g/cm^2^) lower than those by Prodigy (Table 4). The differences between the iDXA and Prodigy regional total body BMD values ranged from −11.6% (arms) to 26.1% (ribs) (Table 4). In particular, for total body BMD values, the difference was strongly dependent on the mean BMD: at high BMD values iDXA showed higher values than Prodigy, whereas at low BMD values the opposite was found (Figure 2). After the correction equations were applied, difference in BMD values between the Prodigy and iDXA devices was negligible. The variables included in the final multiple regression models were based on statistical significance, *r* ^2^ (coefficient of determination), and SEE values (Appendices A and B). Height, weight, or BMI were not included in the final models (*p* > 0.05) for femoral neck, total hip, lumbar spine, and total body BMD regions of interest (ROIs).

## 5. Discussion

The present study, based on a sample of 62–72 women, indicated systematic differences between the GE Healthcare Lunar Prodigy and iDXA DXA devices. BMD scatter plots (Prodigy versus iDXA) showed a close linear relationship over the entire range of BMD values. However, in all ROIs the regression slope was significantly different from unity demonstrating the need for cross-calibration. The BMD values measured using iDXA were 1.5% higher at the lumbar spine (L2–L4), 0.5% higher at the femoral neck, and 0.9% higher at the total hip, whereas total body BMD measurement values were −1.3% lower, compared to those obtained with Prodigy.

Three phantoms were measured with both instruments and a variable BMD disagreement up to 1.4% (from −0.4% to 1.0%) between the instruments was registered. In comparison of* in vitro* and* in vivo* results the disagreement between ESP phantom measurements and* in vivo* spinal BMD and femoral neck values was ±0.5%. The discrepancy between Hologic phantom results and* in vivo* measurements ranged from 0.9% (femoral neck) up to 1.9% (spine). Furthermore, the closest agreement between the DXA devices was observed in the Lunar Phantom (0.4%) and the* in vivo* femoral neck BMD values (i.e., 0.5%), but not at spine with a disagreement of 0.9%. Calibration using ESP phantom may agree more closely with the* in vivo* cross-calibration results, as compared to use of Lunar aluminum phantom with straight edges [12]. Calibrations between different densitometers, as based on previous and present* in vivo* and* in vitro* measurements, may agree [15]. However, a cross-calibration between two DXA modalities based on only phantom measurements can also be inaccurate, especially at hip and total body ROIs [15]. According to ISCD phantom-based cross-calibration is adequate after hardware change or after replacing the DXA system with the same model from the same manufacturer. Instead,* in vivo* cross-calibration is necessary if the old DXA system is replaced with a different model from the same or different manufacturer [1].

Cross-calibration between the devices is essential as the mean systematic differences between instruments may exceed the annual biological BMD changes [1]. Typically, differences of below 1% are encountered between similar or different devices from the same manufacturer [2, 16, 17]. In the present study, iDXA measured BMD values at the lumbar spine (L2–L4), femoral neck, and total hip were higher than those measured with Prodigy (i.e., 1.5%, 0.5%, and 0.9%, resp.). In contrast, previous studies have reported that BMD_iDXA_ values were lower than BMD_Prodigy_ values at the lumbar spine (ranging from −0.25% to −1.2%), femoral neck (ranging from −0.7% to −2.0%), and total hip (ranging from −0.1% to −0.2%) ROIs [4, 6, 7]. As an exception Choi et al. measured BMD_iDXA_ values to be 0.3% higher than BMD_Prodigy_ values at the total hip ROI [4]. Thus, compared to previous results [4, 6, 7], the discrepancy could be 2.7%, 2.5%, and 1.1% at spine [4], femoral neck [4], and total hip [7], respectively. Importantly, linear correction equations between iDXA and Prodigy, as derived in these previous cross-calibration studies [4, 6, 7] and implemented in the present study, would have increased the disagreement, especially at the femoral neck, the total hip, and the lumbar spine ROIs, between our two DXA devices. Furthermore, no significant correlations were observed between BMD_Prodigy_ and BMD_iDXA_ differences and mean BMD values of the two devices in femoral neck, total hip, and lumbar spine ROIs [4]. According to ISCD, correction equations are needed if the difference of two densitometers exceeds 1% [1]. In the present study, the femoral neck BMD values differed by 0.5% between Prodigy and iDXA, indicating no true need for cross-calibration. However, after applying correction coefficients the difference between these two densitometers was negligible. A 0.005 g/cm^2^ difference of femoral neck BMD values between the Prodigy and iDXA devices found in the present study corresponds to a typical mean femoral neck bone loss during a one-year period [18].

In the present study, the average total body BMD measurement values acquired with iDXA were −1.3% lower than those with Prodigy and are, thus, in accordance with previous studies (ranging from −1.4 to −3.5%) [5, 8]. Still, the maximum discrepancy was 2.2% between the previous [5] and present study. The total body BMD values, especially at high bone density levels, were considerably higher with iDXA than with Prodigy [8]. Furthermore, significantly higher differences occurred in regional BMD values between the two devices. For example, mean BMD values of iDXA in arms ROI and in legs ROI were −11.6% and −6.4% lower, respectively, as compared to Prodigy. The differences between the devices in regional BMD values in arms (−13.9% [8] versus −11.6% (present study)), legs (−4.5% versus −6.4%), pelvis (−11.9% versus −11.0%), and ribs (26.1% versus 26.1%) ROIs were nearly similar to those found earlier. In contrast, in the present and previous [8] studies iDXA overestimated and underestimated total body spine BMD by 4.2% and by −3.3%, respectively, when compared to Prodigy.

We also calculated SEE values to analyze the accuracy of our linear predictions. The present SEE values were similar as presented earlier at femoral neck, at lumbar spine [5, 6], at total body [7], and at total hip [6]. Total hip SEE values were even slightly lower than previously [5]. Importantly, the larger the SEE, is the more difficult it is to detect true BMD changes after a scanner change. Limits of agreement for BMD values are compatible in most ROIs or slightly wider in some ROIs compared to previous reports [4, 7].

There are obvious reasons for some differences in the results of present and previous studies [4–7]. As BMD values and anthropometrical variables may differ between Caucasian and Asian people [19–21], the correction equations obtained from a study of Asian people [4] or multiethnic subjects [5] may not be implemented for the present study population. However, it is not possible to address whether this BMD discrepancy [4] is mainly due to variation in output of individual DXA instruments or to some extent due to ethnicity. Indeed, up to 5% differences may exist between the same types of devices from the same manufacturer in worst-case scenario [22]. Further, both BMD values and hip geometry parameters differ significantly by gender [23]. Previous cross-calibration studies between Prodigy and iDXA have included both genders [4–7]. Gender affects cross-calibration at hip [4, 6] and at spine [24]. Earlier, separate correction equations between Prodigy and iDXA were derived for both male and female subjects in a single study [5]. As only women are included in the OSTPRE study [9], correction equations derived from women only may be more appropriate in the context of OSTPRE study. However, ISCD makes no remark whether gender should be taken into account during cross-calibration studies [1]. Also different software versions may affect BMD calibration level and produce systematic differences or errors [25, 26]. In our research strategy we are conservative towards changes in software versions; however, they are inevitable in longitudinal studies such as OSTPRE. To evaluate any drift or change in the measurement accuracy during the study period, quality control scans on spine phantom (L2–L4) were performed along the guidelines of the manufacturer. Thus, the present results apply only to Caucasian women as well as to these two particular DXA devices and software programs examined. Furthermore, all OSTPRE study subjects have been measured with this same set of DXA devices by the same trained staff, thus reducing the DXA device uncertainty compared to that in the multicenter studies with different DXA devices from the same manufacturer or even from different manufacturers [27]. Importantly, the present study population exceeds the ISCD recommended 50 individuals for a cross-calibration study [1].

Deviation of BMD results between two devices suggests significant variations in the DXA technology and possibly, for example, in edge detection algorithms. Through the developments, the image resolution of iDXA is superior to that of Prodigy [5]. Body composition measurements also differ significantly between iDXA and Prodigy [5, 28, 29] and this disagreement is affected by gender [5]. Inclusion of anthropometrical measurements may improve the agreement of BMD measurements between DXA densitometers [2, 3]. Indeed, inclusion of the femur thickness and percentage femur fat was earlier found to improve the agreement between iDXA and Prodigy in femoral neck and total hip BMD, respectively [7]. Spinal BMD agreement was only slightly but still significantly affected by subject height, but not BMI or weight [4]. In the present study, inclusion of subject weight, height, or BMI did not improve the agreement of BMD measurements between the devices at the femoral neck, total hip, lumbar spine, or total body ROIs.

In the present study, both linear and Deming regression were used to analyze the data. Both methods yielded similar results (data not shown). The linear regression has been criticized as it assumes no random error in the dependent variable and may underestimate the slope of the true linear relationship [13]. However, the original input data seems to have more influence on the reliability of the linear regression data than the particular regression procedure applied [30]. In Deming regression we compare true values of two variables (with no experimental error); that is, the regression takes into account the random error of both the dependent and independent variables, typical situation in BMD measurements of the same subjects by two instruments. However, different subjects are measured in other iDXA studies that need implementation of the correction equations. Then, we have only experimental iDXA values in use, leading to obvious contradiction. To enable easier comparison and continuation with our earlier and future DXA studies, the results based on linear regression are presented in the tables.

In conclusion, iDXA measured higher BMD values than Prodigy at spine (1.5%), at femoral neck (0.5%), and at total hip (0.9%) ROIs, whereas total BMD values were lower (−1.3%), respectively. At these ROIs the differences in BMD values of the two devices were found to be independent of the anthropometrical parameters. The differences in total body BMD values were dependent on the mean BMD. Differences between the two devices were negligible when* in vivo* correction coefficients were applied. The current results apply to these two particular DXA devices and software programs used in the study. Importantly, the present results differed significantly from the results of previously published cross-calibration studies between iDXA and Prodigy.

## Figures and Tables

**Table 1:** Mean (SD) values and short-term precisions (CV% = (SD/mean) × 100%) for repeated phantom (*in vitro*) measurements at L2–L4 (*n* = 10), as measured by GE Healthcare Lunar Prodigy and iDXA. Lunar, ESP, and Hologic phantoms were included. Bone mineral values differed significantly between Prodigy and iDXA. Discordant results were obtained with different phantoms.

Phantom		Prodigy			iDXA		
		BMD^a^	BMC^b^	AREA^c^	BMD^a^	BMC^b^	AREA^c^
Lunar	Mean (SD)	1.198 (0.005)^d^	61.47 (0.16)^d^	51.34 (0.12)^d^	1.203 (0.002)	61.24 (0.10)	51.76 (0.04)
	CV%	0.38	0.25	0.23	0.13	0.17	0.08

ESP	Mean (SD)	1.083 (0.005)^d^	30.30 (0.20)^d^	27.97 (0.17)^d^	1.095 (0.005)	31.22 (0.15)	28.51 (0.15)
	CV%	0.50	0.67	0.60	0.42	0.49	0.51

Hologic	Mean (SD)	1.165 (0.004)^d^	47.06 (0.19)^d^	40.40 (0.08)^d^	1.161 (0.003)	46.74 (0.12)	40.27 (0.06)
	CV%	0.34	0.39	0.20	0.26	0.26	0.16

^a^Bone mineral density (g/cm^2^), ^b^bone mineral content (g), and ^c^area (cm^2^) of the L2–L4 region of interest.

^d^Paired samples *t*-test Prodigy versus iDXA (*p* < 0.050).

**Table 2:** Characteristics of the study population (*n* = 72).

	Mean (SD)	Range
Age (yr)	42.2 (16.4)	21–72
Height (cm)	163.6 (5.4)	146–174
Weight (kg)	67.7 (12.4)	52–126
BMI (kg/m^2^)^a^	25.3 (4.7)	18.3–48.5

^a^Body mass index (BMI).

**Table 3:** Mean (SD) values and linear correlation coefficients (*r*) of the *in vivo* dual-energy X-ray absorptiometry (DXA) measurements with Prodigy and iDXA (*n* = 72). Bland and Altman analysis results with relative mean differences *d*%^a^ as well as limits of agreement [*d*  ±  (1.96 × SD)]. Simple linear regression analysis of Prodigy (dependent) versus iDXA (independent) BMD data with standard error (SE) and standard errors of estimates (SEE). Systematic BMD differences were observed between Prodigy and iDXA. After linear regression correction equations were applied the differences were negligible.

BMD ROI	Prodigy mean (SD)	iDXA mean (SD)	*r*	*d*%^a^	Intercept (SE)^c^	Slope (SE)^c^	SEE	SEE (%)^d^	Limits of agreement
Spine L2–L4	1.169 (0.145)	1.186 (0.149)^b^	0.990	1.5		0.985 (0.002)	0.021	1.8	−0.025 to 0.059
Femoral neck	0.941 (0.129)	0.946 (0.128)^b^	0.986	0.5		0.995 (0.003)	0.022	2.3	−0.037 to 0.048
Ward's triangle	0.761 (0.145)	0.758 (0.148)	0.985	−0.4		1.002 (0.004)	0.026	3.4	−0.05 to 0.05
Trochanter	0.800 (0.102)	0.806 (0.108)^b^	0.987	0.7	0.044 (0.015)	0.939 (0.019)	0.017	2.1	−0.030 to 0.040
Shaft	1.162 (0.164)	1.180 (0.168)^b^	0.993	1.5		0.985 (0.002)	0.020	1.7	−0.022 to 0.058
Total hip	0.993 (0.126)	1.002 (0.129)^b^	0.995	0.9		0.990 (0.002)	0.013	1.3	−0.016 to 0.034

^a^Formula for relative mean difference (*d*%): [(iDXA − Prodigy) × 100/Prodigy].

^b^Significantly different (*p* < 0.05) mean difference (*d* = Prodigy − iDXA, paired *t*-test or nonparametric Wilcoxon signed-rank test in Ward' triangle BMD, BMC, or area ROIs).

^c^Correction equation: Prodigy (BMD) = Slope × iDXA (BMD) + intercept.

^d^Formula for SEE (%): [SEE × 100/((Prodigy + iDXA)/2)].

**Table 4:** Mean (SD) values and linear correlation coefficients (*r*) of the *in vivo* dual-energy X-ray absorptiometry (DXA) measurements with Prodigy and iDXA (*n* = 62). Bland and Altman analysis results with relative mean differences *d*%^a^ as well as limits of agreement [*d*  ±  (1.96 × SD)]. Simple linear regression analysis of Prodigy (dependent) versus iDXA (independent) BMD data with standard error (SE) and standard errors of estimates (SEE). Regional total body BMD values differed considerably between Prodigy and iDXA. BMD discrepancy was smaller but significant at total body region of interest. After linear regression correction equations were applied the differences were negligible.

	Prodigy mean (SD)	iDXA mean (SD)	*r*	*d*%^a^	Intercept (SE)^c^	Slope (SE)^c^	SEE	SEE (%)^d^	Limits of agreement
Arms	0.936 (0.091)	0.827 (0.093)^b^	0.848	−11.6	0.251 (0.056)	0.828 (0.067)	0.049	5.5	−0.208 to −0.009
Trunk	0.891 (0.068)	0.932 (0.095)^b^	0.945	4.6	0.263 (0.028)	0.674 (0.030)	0.022	2.5	−0.034 to 0.116
Spine	0.995 (0.102)	1.037 (0.112)^b^	0.922	4.2	0.128 (0.047)	0.836 (0.045)	0.040	3.9	−0.044 to 0.128
Pelvis	1.101 (0.096)	0.979 (0.114)^b^	0.928	−11.0	0.331 (0.040)	0.785 (0.041)	0.036	3.5	−0.206 to −0.036
Legs	1.233 (0.099)	1.154 (0.104)^b^	0.979	−6.4	0.162 (0.029)	0.929 (0.025)	0.020	1.7	−0.121 to −0.037
Total body	1.162 (0.078)	1.146 (0.095)^b^	0.962	−1.3	0.257 (0.033)	0.789 (0.029)	0.021	1.9	−0.073 to 0.041

^a^Formula for relative mean difference (*d*%): [(iDXA − Prodigy) × 100/Prodigy].

^b^Significantly different (*p* < 0.05) mean difference (*d* = Prodigy − iDXA, paired *t*-test or nonparametric Wilcoxon signed-rank test in Ward's triangle BMD, BMC, or area ROIs).

^c^Correction equation: Prodigy (BMD) = Slope × iDXA (BMD) + intercept.

^d^Formula for SEE (%): [SEE × 100/((Prodigy + iDXA)/2)].

## Appendix

### A. Bone Mineral Density Correction Equations Based on Simple Regression Analyses between the Prodigy and iDXA Devices

#### A.1. Simple Correction Coefficients (Lumbar Spine and Hip ROIs)

Lumbar spine (L2–L4):
  Prodigy BMD = 0.9853 × iDXA BMD,
  Prodigy BMC = 0.9881 × iDXA BMC,
  Prodigy AREA = 1.0026 × iDXA AREA.
Lumbar spine (L2-L3):
  Prodigy BMD = 0.9863 × iDXA BMD,
  Prodigy BMC = 0.9571 × iDXA BMC + 1.1534,
  Prodigy AREA = 1.0077 × iDXA AREA.
Lumbar spine (L3-L4):
  Prodigy BMD = 0.9841 × iDXA BMD,
  Prodigy BMC = 0.9867 × iDXA BMC,
  Prodigy AREA = 1.0018 × iDXA AREA.
Femoral neck:
  Prodigy BMD = 0.9946 × iDXA BMD,
  Prodigy BMC = 0.9900 × iDXA BMC,
  Prodigy AREA = 0.9380 × iDXA AREA + 0.2712.
Ward's triangle:
  Prodigy BMD = 1.0023 × iDXA BMD,
  Prodigy BMC = 0.9354 × iDXA BMC + 0.1103,
  Prodigy AREA = 0.9253 × iDXA AREA + 0.1620.
Trochanter:
  Prodigy BMD = 0.9394 × iDXA BMD + 0.0436,
  Prodigy BMC = 0.9679 × iDXA BMC,
  Prodigy AREA = 0.9753 × iDXA AREA.
Femur shaft:
  Prodigy BMD = 0.9846 × iDXA BMD,
  Prodigy BMC = 0.9832 × iDXA BMC,
  Prodigy AREA = 0.9981 × iDXA AREA.
Total hip:
  Prodigy BMD = 0.9904 × iDXA BMD,
  Prodigy BMC = 0.9798 × iDXA BMC,
  Prodigy AREA = 0.9893 × iDXA AREA.

#### A.2. Simple Correction Coefficients (Regional and Total Body ROIs)

Head:
  Prodigy BMD = 0.9112 × iDXA BMD + 0.1448,
  Prodigy BMC = 0.8942 × iDXA BMC + 25.2796,
  Prodigy AREA = 0.9679 × iDXA AREA.
Arms:
  Prodigy BMD = 0.8282 × iDXA BMD + 0.2507,
  Prodigy BMC = 1.0255 × iDXA BMC,
  Prodigy AREA = 0.4823 × iDXA AREA + 149.4271.
Ribs:
  Prodigy BMD = 0.4858 × iDXA BMD + 0.2437,
  Prodigy BMC = 1.0313 × iDXA BMC,
  Prodigy AREA = 1.8529 × iDXA AREA − 131.7785.
Trunk:
  Prodigy BMD = 0.6739 × iDXA BMD + 0.2630,
  Prodigy BMC = 1.0784 × iDXA BMC,
  Prodigy AREA = 1.5614 × iDXA AREA − 309.3132.
Spine:
  Prodigy BMD = 0.8363 × iDXA BMD + 0.1278,
  Prodigy BMC = 1.2697 × iDXA BMC,
  Prodigy AREA = 0.9845 × iDXA AREA + 58.1811.
Pelvis:
  Prodigy BMD = 0.7854 × iDXA BMD + 0.3313,
  Prodigy BMC = 0.9914 × iDXA BMC,
  Prodigy AREA = 0.8757 × iDXA AREA.
Legs:
  Prodigy BMD = 0.9285 × iDXA BMD + 0.1615,
  Prodigy BMC = 1.0819 × iDXA BMC,
  Prodigy AREA = 1.0123 × iDXA AREA.
Total body:
  Prodigy BMD = 0.7894 × iDXA BMD + 0.2569,
  Prodigy BMC = 1.0421 × iDXA BMC,
  Prodigy AREA = 1.0265 × iDXA AREA.

#### A.3. Multivariate Correction Coefficients (BMI, Weight, or Height Was Included in the Final Model, **p** < 0.05)

Ward's triangle:
  Prodigy BMD = 0.9663 × iDXA BMD − 0.0019 × BMI + 0.0776.
  iDXA BMD = 0.9966 × Prodigy BMD,
  iDXA BMC = 1.0066 × Prodigy BMC,
  iDXA AREA = 1.0100 × Prodigy AREA.
Head:
  Prodigy BMD = 0.9311 × iDXA BMD − 0.0029 × height + 0.0019 × weight + 0.4496,
  Prodigy BMC = 0.9088 × iDXA BMC + 1.2608 × BMI − 13.6528.
Arms:
  Prodigy BMD = 0.7913 × iDXA BMD + 0.0041 × height − 0.3964,
  Prodigy AREA = 0.3842 × iDXA AREA + 1.3734 × height − 40.7580.
Ribs:
  Prodigy BMD ribs = 0.4574 × iDXA BMD + 0.0006 × weight + 0.2260.
Trunk:
  Prodigy BMD = 0.6601 × iDXA BMD + 0.0006 × weight + 0.2363.
Spine:
  Prodigy BMD = 0.8571 × iDXA BMD − 0.0027 × BMI + 0.1739,
  Prodigy AREA = 0.8493 × iDXA AREA + 0.6926 × weight + 34.8719.
Pelvis:
  Prodigy BMD = 0.7979 × iDXA BMD + 0.0050 × BMI + 0.1947.
Legs:
  Prodigy BMC = 1.0331 × iDXA BMC − 1.3119 × height + 2.4172 × weight + 94.1616,
  Prodigy AREA = 0.8682 × iDXA AREA + 2.1046 × weight − 37.3970.
Total body:
  Prodigy BMC = 1.0763 × iDXA BMC + 9.0931 × BMI − 306.2178,
  Prodigy AREA = 0.9130 × iDXA AREA + 3.5498 × weight − 9.5166.

### B. Bone Mineral Density Correction Equations Based on Multiple Regression Analyses between the Prodigy and iDXA Devices

#### B.1. Simple Correction Coefficients (Lumbar Spine and Hip ROIs)

Lumbar spine (L2–L4):
  iDXA BMD = 1.0146 × Prodigy BMD,
  iDXA BMC = 1.0114 × Prodigy BMC,
  iDXA AREA = 0.9970 × Prodigy AREA.
Lumbar spine (L2-L3):
  iDXA BMD = 1.0135 × Prodigy BMD,
  iDXA BMC = 1.0060 × Prodigy BMC,
  iDXA AREA = 0.9919 × Prodigy AREA.
Lumbar spine (L3-L4):
  iDXA BMD = 1.0157 × Prodigy BMD,
  iDXA BMC = 1.0126 × Prodigy BMC,
  iDXA AREA = 0.9977 × Prodigy AREA.
Femoral neck:
  iDXA BMD = 1.0050 × Prodigy BMD,
  iDXA BMC = 1.0095 × Prodigy BMC,
  iDXA AREA = 1.0047 × Prodigy AREA.
Ward's triangle:
  iDXA BMD = 0.9966 × Prodigy BMD,
  iDXA BMC = 1.0066 × Prodigy BMC,
  iDXA AREA = 1.0100 × Prodigy AREA.
Trochanter:
  iDXA BMD = 1.0071 × Prodigy BMD,
  iDXA BMC = 1.0314 × Prodigy BMC,
  iDXA AREA = 1.0241 × Prodigy AREA.
Femur shaft:
  iDXA BMD = 1.0153 × Prodigy BMD,
  iDXA BMC = 1.0167 × Prodigy BMC,
  iDXA AREA = 1.0016 × Prodigy AREA.
Total hip:
  iDXA BMD = 1.0095 × Prodigy BMD,
  iDXA BMC = 1.0204 × Prodigy BMC,
  iDXA AREA = 1.0107 × Prodigy AREA.

#### B.2. Simple Correction Coefficients (Regional and Total Body ROIs)

Head:
  iDXA BMD = 1.0270 × Prodigy BMD,
  iDXA BMC = 1.0608 × Prodigy BMC,
  iDXA AREA = 1.0327 × Prodigy AREA.
Arms:
  iDXA BMD = 0.8838 × Prodigy BMD,
  iDXA BMC = 0.9739 × Prodigy BMC,
  iDXA AREA = 1.1105 × Prodigy AREA.
Ribs:
  iDXA BMD = 1.7060 × Prodigy BMD − 0.2799,
  iDXA BMC = 0.6344 × Prodigy BMC + 65.8222,
  iDXA AREA = 0.3116 × Prodigy AREA + 140.3294.
Trunk:
  iDXA BMD = 1.3247 × Prodigy BMD − 0.2484,
  iDXA BMC = 0.6944 × Prodigy BMC + 167.3488,
  iDXA AREA = 0.3638 × Prodigy AREA + 417.3535.
Spine:
  iDXA BMD = 1.0420 × Prodigy BMD,
  iDXA BMC = 0.7831 × Prodigy BMC,
  iDXA AREA = 0.4377 × Prodigy AREA + 70.9764.
Pelvis:
  iDXA BMD = 1.0961 × Prodigy BMD − 0.2269,
  iDXA BMC = 0.7655 × Prodigy BMC + 71.8889,
  iDXA AREA = 0.4874 × Prodigy AREA + 172.6118.
Legs:
  iDXA BMD = 1.0318 × Prodigy BMD − 0.1181,
  iDXA BMC = 0.9233 × Prodigy BMC,
  iDXA AREA = 0.7966 × Prodigy AREA + 138.6117.
Total body:
  iDXA BMD = 1.1726 × Prodigy BMD − 0.2160,
  iDXA BMC = 0.8560 × Prodigy BMC + 249.3750,
  iDXA AREA = 0.6022 × Prodigy AREA + 764.6783.

#### B.3. Multivariate Correction Coefficients (BMI, Weight, or Height Was Included in the Final Model, **p** < 0.05)

Ward's triangle:
  iDXA BMD = 1.0022 × Prodigy BMD + 0.0008 × weight − 0.0564.
Trochanter:
  iDXA BMC = 0.9956 × Prodigy BMC + 0.0251 × BMI − 0.3063,
  iDXA AREA = 0.9584 × Prodigy AREA + 0.0230 × BMI + 0.1738.
Head:
  iDXA BMD = 1.0373 × Prodigy BMD + 0.0036 × height − 0.0020 × weight − 0.4834,
  iDXA BMC = 1.0662 × Prodigy BMC − 1.4831 × BMI + 34.0991.
Arms:
  iDXA BMD = 0.9347 × Prodigy BMD − 0.0031 × height + 0.4656,
  iDXA AREA = 1.2162 × Prodigy AREA − 2.1627× BMI + 19.5001.
Ribs:
  iDXA BMC = 0.5811 × Prodigy BMC + 0.8734 × weight + 17.8186,
  iDXA AREA = 0.2834 × Prodigy AREA + 0.8194 × weight + 94.2012.
Trunk:
  iDXA AREA = 0.2688 × Prodigy AREA + 2.7170 × height + 1.7792 × weight − 71.5153.
Spine:
  iDXA BMD = 1.0003 × Prodigy BMD + 0.0037 × BMI − 0.0501.
Pelvis:
  iDXA BMD = 1.1183 × Prodigy BMD + 0.0022× height − 0.0021 × weight − 0.4715,
  iDXA BMC = 0.7145 × Prodigy BMC + 1.3401 × height − 132.8884,
  iDXA AREA = 0.2891 × Prodigy AREA + 1.4589 × height + 0.9313 × weight − 76.3318.
Legs:
  iDXA BMC = 0.9109 × Prodigy BMC + 2.0435 × height − 1.9780 × weight − 190.9683,
  iDXA AREA = 0.7992 × Prodigy AREA + 2.1033 × height − 1.1282 × weight − 132.8240.
Total body:
  iDXA BMC = 0.7893 × Prodigy BMC + 6.7959 × height − 705.1012,
  iDXA AREA = 0.3903 × Prodigy AREA + 11.4165 × height − 671.5098.
